# Supplementary material for: Long Non-Coding RNA LINC02802 Regulates In Vitro Sprouting Angiogenesis by Sponging microRNA-486-5p
Source: Int J Mol Sci. 2022 Jan 31;23(3):1653. doi: 10.3390/ijms23031653 (PMC8836176; doi:10.3390/ijms23031653)
Supplement: Supplementary file 1 [file ijms-23-01653-s001.zip › SUPPLEMENTARY/Figure supplementary1.pdf]

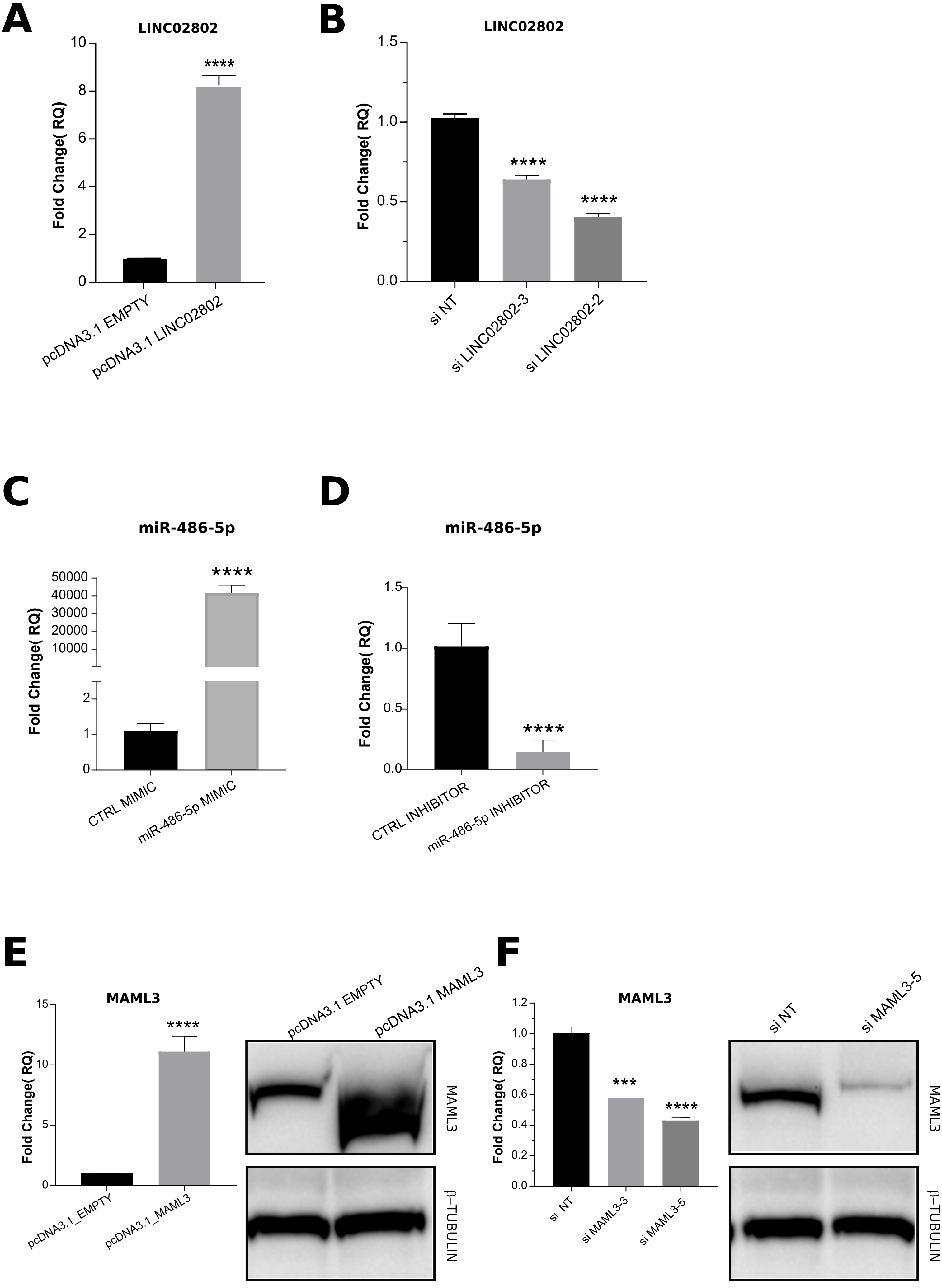
A
LINC02802
Fold Change(RQ)
0
2
4
6
8
10
****
pcDNA3.1 EMPTY
pcDNA3.1 LINC02802
B
LINC02802
Fold Change(RQ)
0.0
0.5
1.0
1.5
****
****
si NT
si LINC02802-3
si LINC02802-2
C
miR-486-5p
Fold Change(RQ)
0
1
2
10000
20000
30000
40000
50000
****
CTRL MIMIC
miR-486-5p MIMIC
D
miR-486-5p
Fold Change(RQ)
0.0
0.5
1.0
1.5
****
CTRL INHIBITOR
miR-486-5p INHIBITOR
E
MAML3
Fold Change(RQ)
0
5
10
15
****
pcDNA3.1_EMPTY
pcDNA3.1_MAML3
pcDNA3.1 EMPTY
pcDNA3.1 MAML3
MAML3
β–TUBULIN
F
MAML3
Fold Change(RQ)
0.0
0.2
0.4
0.6
0.8
1.0
1.2
***
****
si NT
si MAML3-3
si MAML3-5
si NT
si MAML3-5
MAML3
β–TUBULIN

Supplementary Figure S1: Validation of efficiency of over-expression and knockdown. (A) The effectiveness of over-expression of LINC02802 and (B) knockdown confirmed by real-time PCR. Data are representative of n = 3 experiments; ****, p<0.0001confirmed by RT-qPCR (C-D) The effectiveness of over-expression and knockdown of mir-486-5p respectively. Data are represented as mean ± SEM from n = 3 experiments; ****, p<0.0001; ***, p<0.001; **, p<0.01; *, p<0.05. (E) RT-qPCR and western blot analisys of MAML3 expression in ECs transfected with pcdna3.1_MAML3. (F) RT-qPCR and western blot analisys of MAML3 expression in ECs transfected with siRNA against MAML3. Si MAML3-5 resulted in a lower level of MAML3 expression was selected for the following validations. Data are represented as mean ± SEM from n = 3 experiments; ****, p<0.0001. Blot are representative of 3 experiments done with similar results.
